# Roles of Silk Fibroin on Characteristics of Hyaluronic Acid/Silk Fibroin Hydrogels for Tissue Engineering of Nucleus Pulposus

**DOI:** 10.3390/ma13122750

**Published:** 2020-06-17

**Authors:** Tze-Wen Chung, Weng-Pin Chen, Pei-Wen Tai, Hsin-Yu Lo, Ting-Ya Wu

**Affiliations:** 1 Department of Biomedical Engineering, National Yang-Ming University, Taipei 11221, Taiwan; ylno02ekil@yahoo.com.tw (P.-W.T.); catfish19930628@gmail.com (H.-Y.L.); wuyaya0317@gmail.com (T.-Y.W.); 2 Center for Advanced Pharmaceutical Science and Drug Delivery, National Yang-Ming University, Taipei 11221, Taiwan; 3 Department of Mechanical Engineering, National Taipei University of Technology, Taipei 10608, Taiwan; 4 Additive Manufacturing Center for Mass Customization Production, National Taipei University of Technology, Taipei 10608, Taiwan

**Keywords:** silk fibroin, hyaluronic acid, Tyrosine, viscoelastic modulus of HS-IPN hydrogels, hBMSC differentiations, nucleus pulposus

## Abstract

Silk fibroin (SF) and hyaluronic acid (HA) were crosslinked by horseradish peroxidase (HRP)/H_2_O_2_, and 1,4-Butanediol di-glycidyl ether (BDDE), respectively, to produce HA/SF-IPN (interpenetration network) (HS-IPN) hydrogels. HS-IPN hydrogels consisted of a SF strain with a high content of tyrosine (e.g., strain A) increased viscoelastic modules compared with those with low contents (e.g., strain B and C). Increasing the quantities of SF in HS-IPN hydrogels (e.g., HS7-IPN hydrogels with weight ratio of HA/SF, 5:7) increased viscoelastic modules of the hydrogels. In addition, the mean pores size of scaffolds of the model hydrogels were around 38.96 ± 5.05 μm which was between those of scaffolds H and S hydrogels. Since the viscoelastic modulus of the HS7-IPN hydrogel were similar to those of human nucleus pulposus (NP), it was chosen as the model hydrogel for examining the differentiation of human bone marrow-derived mesenchymal stem cell (hBMSC) to NP. The differentiation of hBMSC induced by transforming growth factor β3 (TGF-β3) in the model hydrogels to NP cells for 7 d significantly enhanced the expressions of glycosaminoglycan (GAG) and collagen type II, and gene expressions of aggrecan and collagen type II while decreased collagen type I compared with those in cultural wells. In summary, the model hydrogels consisted of SF of strain A, and high concentrations of SF showed the highest viscoelastic modulus than those of others produced in this study, and the model hydrogels promoted the differentiation of hBMSC to NP cells.

## 1. Introduction

Hydrogels can be produced by crosslinking polymers to form interpenetration network (IPN) with varying mechanical properties [1,2] for tissue engineering such as cardiac tissue repairs [3], controlling the fates of stem cells [1], and drug delivery, etc. [4]. Since hydrogels are highly permeable to nutrients and water-soluble metabolites, they can support cell growth and proliferation which are suitable for tissue engineering (TE). Hydrogels for TE usually consist of synthetic polymers, such as polyurethane and polyvinyl alcohol (PVA) [5,6], or natural polymers, such as HA and collagen [7,8].

HA is a natural glycosaminoglycan with carboxylic groups; it is an important component of the extracellular matrices (ECM) in various tissues and play important roles in cell proliferation and migration [9]. For instance, the interactions of HA in a cardiac patch and CD44 of BMSC enhanced cardiac differentiations of BMSC in both cardiac gene and proteins expressions [10]. In addition, various methods to prepare HA-based hydrogels including oxidized-HA or methacrylated-HA have been investigated for TE of ECM of NP [11,12,13], respectively.

Various of SF-based membranes, scaffolds or hydrogels have been extensively studied for the applications of TE because of its favorable biological responses, such as weak antigenic effects and inflammatory responses in-vivo [9,14,15,16]. For example, SF/HA patches laden with hBMSC promoted cardiac repair in a rat myocardial infarction (MI) model [10,16]. Developing suitable mechanical properties for hydrogels is also important to enhance cell proliferations and hBMSC differentiations for using various TE [17,18]. In this regard, the crosslinking tyrosine in SF to produce di-tyrosine bonds by HRP/H_2_O_2_ enzymatic reactions produced silk elastomers with stiffness that are varied from 0.2 to 10 kPa [17]. However, the stiffness of the aforementioned SF hydrogels was not suitable for the needs of some tissues such as human NP. Although the influences of molecular weights of SF on mechanical properties of SF hydrogels have been reported [17], the influence of total tyrosine contents in SF on the mechanical properties of SF-based hydrogels has not been investigated.

The intervertebral disc (IVD) absorbs shocks by transferring and dissipating loads to the ECM within the superior and inferior discs. IVD degeneration generally causes lower back pain, which is a common health problem. [7,18]. Currently, clinical treatments, such as spinal fusion and partial or total disc replacement cannot fully restore or maintain IVD structures and functions [7,18,19]. Since disc degeneration originates in nucleus pulposus (NP) regions, tissue engineering NP, that may mimic the structure of native NP tissue and possibly fully restore the functionality of healthy IVD discs, is great needed [18,19].

Various biomaterials have been investigated for TE of NP. They are: A. carbohydrate polymers including HA, dextran, chitosan and carboxymethylcellulose (CMC) to produce various hydrogels such as oxidized-HA or methacrylated-HA [11,12,13], dextran-chitosan-teleostean and methacrylated CMC hydrogels, respectively [7,18,19]; B. proteins including Type II collagen hydrogels, and laminin-based hydrogels, respectively [20,21]; C. hybrid of carbohydrate polymers and proteins, such as crosslinked oxidized HA/gelatin [12] and Type II collagen with a low molecular weight of HA [8,22]. However, the mechanical properties of some of the aforementioned biomaterials were not suitable for TE of NP, which need to be further processed [11,20,21]. For instance, the elastic modulus of the oxidized-HA hydrogels, using adipic acid di-hydrazide (ADH), are much lower than those of the native ECM of the NP [11]. Although hydrogels produced by oxidized HA-gelatin using ADH could improve the elastic modulus of the hydrogels (~11 kPa), the controlling the chemical reactions of HA/gelatin by ADH might not be easily carried out [12]. Recently, dextran-chitosan-teleostean triple-interpenetrating network and methacrylatedC MC hydrogels has been examined in a goat model to support the mechanical functions of degenerative NP [18,19]. However, chitosan and CMC had not yet been approved by the FDA for use in internal organs.

Although the bioactivities of SF or HA/SF patches for cardiac repairs have been shown in-vitro, and in-vivo, respectively [10,16], the mechanical properties for HS-IPN hydrogels, which consisted of various amounts of tyrosine in SF, in terms of vary strains of SF in this study, and the weight ratios of SF to HA have not been investigated. Moreover, the potential of differentiations of induced hBMSC laden-HS-IPN hydrogels to NP cells has not been examined. Although using NHS/EDC to crosslink HA/SF to facilely prepare HA/SF hydrogels was recently reported [23], it was one step of our processes to prepare hydrogels. However, they did not examine the rheological properties of hydrogels. Using HRP/H_2_O_2_ to crosslink tyramine-substitute HA and 2% of SF to produce hydrogels with varying HA contents has also been reported by Raia et al. [24]. HA and SF-IPN hydrogels produced by them are configured by di-tyramine bonds in HA crosslinked network, di-tyrosine bonds in SF crosslinked network and tyramine-tyrosine bonds in HA/SF-IPN. The tyramine-substitute HA needed to be complexly and chemically synthesized for the research study, which was not a commercially available biomaterial.

**Scheme 1:**
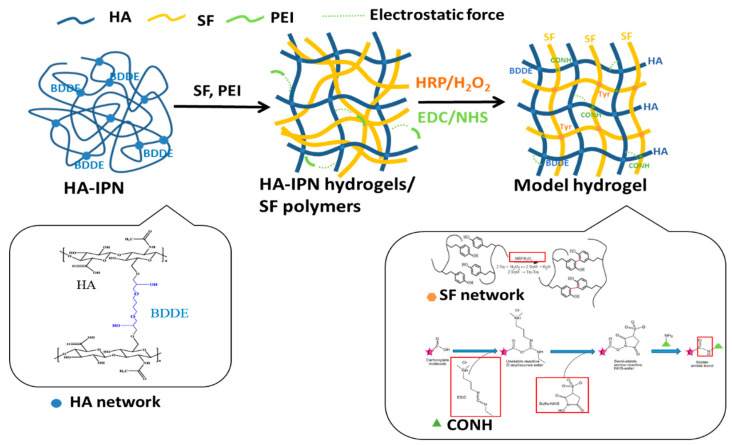
The schematic procedures to develop HS-IPN hydrogel; HA polymer chains were first crosslinked by BDDE reagent for several hours to produce HA crosslinked network hydrogel (blue dots); SF and a small quantity of PEI (polyethyleneimine) were added into the gel and well blended. SF polymer chains were then crosslinked by HRP/H_2_O_2_ enzymatic reactions in HA crosslinked network hydrogel to produce large quantities of di-tyrosine bonds, make SF crosslinked hydrogel, (brown dots or Tyr in the model hydrogels) and yield HS-IPN hydrogel. HS-IPN hydrogel and PEI were further crosslinked using EDC/NHS reagents to produce many amide bonds to stabilize the hydrogels.

To produce HS-IPN hydrogels, HA-crosslinked network hydrogels were first produced by using BDDE solutions to crosslink HA polymers (Scheme 1). The influences of varying strains of SF obtained from *B. mori* cocoons and, at a fixed quantities of HA, varying amounts of SF (expressed in wt. ratios of 5/1~5/7 for HA/SF, respectively) were crosslinked using HRP/H_2_O_2_ reactions to produce HS-IPN hydrogels. To further increase in-vitro stability of hydrogels, the carboxyl acids of HA in HS-IPN hydrogels were crosslinked with amine groups of added polyethyleneimine (PEI) and HS-IPN using *N*-(3-Dimethyl-amino-propyl)-*N*′-ethylcarbodiimide hydrochloride)/*N*-Hydroxy-succinimide (EDC/NHS) reagents (Scheme 1). The differentiation of hBMSC laden on HS-IPN hydrogels, induced using TGF-β3 to NP cells were examined for TE of NP.

## 2. Materials and Methods

### 2.1. Fabrication of Interpenetrating Network HA-SF Hydrogels

Various strains, strain A, B and C herein, of *B. mori* cocoons were the products of artificially cross breeding specific strains of *B. mori* to produce varying toughness of SF that might vary amino acid configurations of SF, including tyrosine contents. Each strain of *B. mori* was well-bred to control the quality of SF, including low variations in amino acid configurations of SF and its cocoon was gifted from MDARES (Miaoli Agricultural Research and Extension Station, Council of Agriculture, Executive Yuan, Miaoli, Taiwan). In addition, the stain A of SF was used in this study without being further specified.

Solution of SF (MW ~ 185 kDa) of strain A was prepared as described in early reports, such as degumming, de-solving in 9.3 M LiBr and removal of Li^+^ using dialysis with DI water that were published by the authors’ laboratory [10,16]. Briefly, silk cocoons were boiled in 0.02 M Na_2_CO for 0.5 h and then rinsed thoroughly in D.I. water to extract the glue-like sericin proteins from silk fibroin, degumming procedures. The extracted SFs were then dissolved in 9.3 M LiBr solution at 60 °C for 4 h to yield a 20% (w/v) solution, which was then dialyzed against D.I. water using a dialysis membrane (MWCO 6000) (Spectra/Por 1, Repligen Co., Waltham, MA, USA) at room temperature to remove salt for 48 h [10,16].

The MW of SF of strain A, B and C were determined by a SDS-PAGE method (Vertical Electrophoresis System, XCell SureLock^TM^ Mini-Cell, Invitrogen, Waltham, MA, USA) with 150 V for 60 min. The bands of samples and molecular weight ladder (Himark Pre-Stained, Invitrogen, Waltham, MA, USA) were stained by a Coomassie Brilliant Blue R-250 (Sigma Corp., St. Louis, MO, USA). The images of the bands of the samples and standard molecular weight ladder were analyzed by an Image J (Windows version, Java 1.6.0-45, 32 bit mode, NIH, Bethesda, MD, USA). For amino acids analysis, 0.4 mg of dried SF samples (e.g., strand A) were hydrolyzed by 6 N HCL at 115 CC for 24 h. The analysis of amino acids of various samples were conducted by an amino acid analyzer (Hitachi L-8900, Tokyo, Japan).

To prepare HA hydrogel (H gel), HA (MW: 200 kDa, Lifecore Biomedical Inc., Chaska, MN, USA) was dissolved in NaOH at a concentration of 10%. 1,4-Butanediol diglycidyl ether (BDDE, Sigma Corp., St. Louis, MO, USA) was used for cross-linking reactions that was conducted at 37 °C for 6–8 h. The pH value of the solution was then adjusted to about 7.0 by adding HCl solution to terminate the crosslinking reaction [25,26]. After the HA-BDDE crosslinking reaction had been terminated, 0.15 mM PEI (polyethyleneimine, Mw. 25 KDa, Sigma, St. Louis, MO, USA), an appropriate amount of H_2_O_2_ (about 0.15 mM) and horseradish peroxidase (50 U/mL) (HRP, Sigma, St. Louis, MO, USA) and 5% SF solution were added to the H gels and well blended. SF in the aforementioned solutions were crosslinked and gradually turned to form the second interpenetration network, SF IPN-gels, because large quantities of di-tyrosine bonds of SF were produced by the reactions of HRP/H_2_O_2_, the enzyme reactions, in H gels for about 1 h at 37 °C [27].

To prepare SF hydrogel (S gel), an appropriate amount of H_2_O_2_ (about 0.15 mM) and horseradish peroxidase (50 U/mL) (HRP, Sigma, St. Louis, MO, USA) were added to 5% SF solution SF of strain A to induce enzymatic reaction for 1 h at 37 °C [27]. HS-IPN hydrogels were produced by blending the aforementioned H gels and S gels (Scheme 1).

To stabilize the HS-IPN hydrogels, 0.15 mM PEI in the hydrogels was further crosslinked by EDC/NHS (1:1) (*N*-(3-Dimethyl-amino-propyl)-*N*′-ethylcarbodiimide hydrochloride, C_8_H_17_N_3_ HCL, EDC), Sigma Corp., St. Louis, MO, USA)/(*N*-Hydroxy-succinimide, C_4_H_5_NO_3_, NHS), Fluka Chemical Corp., Rochester, NY, USA) for around 30 min. at temperature lower than 20 °C to produce amide bonds of carboxyl groups of HA with amine groups in PEI and in SF, respectively (Scheme 1). In addition, to examine the influences of varying ratios of SF on viscoelastic properties of HS-IPN hydrogels, the weight ratios of HA (4% at final) and SF in the hydrogels were changed to 5:1, 5:3, and 5:7 (e.g., HS1-IPN, HS3-IPN, HS7-IPN (namely, the model hydrogels)), respectively.

### 2.2. Characterizations of HS-IPN Hydrogels

#### 2.2.1. Attenuated Total Reflectance-Fourier Transform Infrared (ATR-FTIR) Spectra of the Hydrogels

After various HS-IPN hydrogels were freeze-dried at −50 °C, their transmission spectra of the samples were examined using an ATR-FTIR instrument (IRAffinity-1, Shimadzu Co, Kyoto, Japan) with a resolution of 4 cm^−1^ at wave numbers 400–4000 cm^−1^ [10]. Detailed procedures for the measurements can be referred elsewhere. The spectra were analyzed using the built-in standard software package (IRAffinity-1, Shimadzu Co, Kyoto, Japan) [10].

#### 2.2.2. Swelling Ratios of H, S, Crosslinked H/PEI and the Model Hydrogels

To study the swelling ratios of various gels, the samples were placed in phosphate saline (PBS) allowed for unrestricted deformation swelling test. H/PEI crosslinking hydrogels were fabricated after H gels were produced, followed by crosslinked 0.015 mM PEI in the gels by EDC/NHS crosslinking reagent for 30 min. The purpose of producing H/PEI crosslinking hydrogels was to examine swelling property of the gels after amide bonds formation between the carboxyl groups in HA gels and amine groups of PEI. The weights of hydrogels were measured at time of 0, 0.5, 1, 4, 24 and 48 h, W_t_, after they were immersed into PBS for the aforementioned time. Their surfaces were gently wiped before they were weighted. The swelling ratio of a hydrogel was calculated by the following equation while W_net_ was the weight of the hydrogel before immersed into PBS:

(1) $$\mathrm{Swelling}\;\mathrm{ratio}\;\left(\%\right)=\frac{W_{t}\;{-\;W}_{\mathrm{net}}}{W_{\mathrm{net}}}\;\times \;100\%$$

#### 2.2.3. Morphology of HS-IPN Hydrogels

To observe the morphology and the pore structure of HS-IPN hydrogels, all hydrogels were frozen at −50 °C and then at liquid nitrogen, further dried in vacuum for several days, so-called freeze-dry technique, to produce scaffolds. They were cut by a surgical knife to obtain the cross-section for observing the pore size distributions and characterizing the network in the scaffolds. All samples were coated with Pt and imaged with scanning electron microscopy (SEM, JSM-7600F, JEOL, Tokyo, Japan) at an accelerating voltage of 3–10 kV. The pore sizes of gel were calculated by ImagePro software (IPP7.0, Media Cybernetics, Rockville, MD, USA).

### 2.3. Rheological Studies of Vary Compositions of HS-IPN Hydrogels

The viscoelastic properties of various hydrogels were determined using a rheometer with an oscillatory mode (Discovery-HR1, TA Instruments, New Castle, DE, USA) at 37 °C. Oscillation frequency sweep tests using a parallel-plate sensor with a gap of 1 mm and loaded by 0.1 mL of HS-IPN hydrogels were carried out by the rheometer. The elastic modulus (G′) and viscous modulus (G″), phase shift angle (δ) and complex modulus (|G*|) of vary compositions for HS-IPN hydrogels were determined at a fixed strain of 0.01 rad with varying angular frequencies of 1–100 rad/s, as reported in other studies [11,12,28].

#### Compressive Modules of the Model Hydrogels

An MTS Model 858 Bionix Test System (MTS Corp., Eden Prairie, MN, USA) with a 5 kg load cell was used to perform confined compression tests on the porcine NP and l HS7-IPN hydrogels (or the model hydrogels). An acrylic cylinder with a diameter of 10 mm and a height of 30 mm was made and used as the indenter. A hollow, cylindrical acrylic container with an outer diameter of 30 mm, an inner diameter of 10 mm, and a height of 20 mm, was use in the confined compression test. A cavity with a diameter of 25.5 mm and a depth of 2.5 mm was cut into the bottom of the container and a porous plate was placed at the bottom of it. Porcine NP and hydrogel specimens were prepared and used to fill the acrylic container to a height of 5 mm. The confined compression tests were then carried out with five specimens for each material. A preload of 5 N (63.7 kPa) was applied to the indenter for 10 min to obtain a pressure balance on each specimen. In each confined compression test, following the preload phase, the indenter was moved at 0.1 mm/min until a final strain of 5% for the model hydrogels and the porcine NP while that of 15% (large strain compression) performed for the model hydrogels was mainly used for the comparisons with other studies. The load-displacement curve that was thus obtained for each specimen was then converted to a stress–strain (σ–ε) curve. The elastic modulus E was calculated as E = σ/ε for the linear portion of the stress-strain curve.

### 2.4. Cytotoxicity of the Model Hydrogels

To evaluate the biocompatibility of the model hydrogels, MTT (3-(4,5-dimethylthiazol-2-yl)-2,5-diphenyl-tetrazolium bromide), Sigma–Aldrich, St. Louis, MO, USA) assays were performed to test the cytotoxicity of vary ratios of extraction mediums, taken from supernatants, which was obtained by incubation the hydrogels with L929 fibroblasts for 24 h according to the guidelines of international standard organization (ISO) 10993-5 [29]. L929 fibroblasts were purchased from the Bio-resource Collection and Research Center (BCRC, Hsin-Chu, Taiwan) and cultured in Dulbecco’s modified Eagle’s medium that contained 10% horse serum at 37 °C in a 5% CO2 incubator. After L929 cells were cultured with vary volume ratios of extraction mediums to cultural medium (e.g., 1/8, 1/4, 1/2 and 1.0 (or extraction medium only), namely dilution ratios) for 24 h, MTT assay to the cells were carried out to examine the cell viabilities as early report of this lab [29].

### 2.5. Differentiations of Induced hBMSC to NP in the Model Hydrogels

To evaluate the potential of the model hydrogels for NP regeneration, human bone marrow-derived mesenchymal stem cell (hBMSC) from passage 6~8 were used according to our previous study [10]. The density of hBMSC of ${1\;\times \;10}^{7}\;\mathrm{cell}/\mathrm{mL}$ was seeded on the surface layer of a 1.0 cm^2^ the model hydrogels to produce the cell-laden hydrogels. The viability of hBMSC in the model hydrogels after 3 d of cultivation were stained with Live/Dead or Viability/Cytotoxicity kit (Invitrogen, Waltham, MA, USA) according to the manual instructions. Live/Dead stain of the cells was observed by a laser confocal scanning microscopy (LCSM) (Olympus FV1000, Olympus Corp., Tokyo Japan) [10].

To induce the chondrogenesis of hBMSC in the model hydrogels, after the cell proliferations in the hydrogels for 3 d, the culture medium was changed to chondrogenic differentiation medium containing TGF-β3 which was purchased from Lonza Corp. (Lonza, Gaithersburg, MD, USA). The differentiation medium was changed every two days, and followed by culture for 7 d and 14 d, respectively, for chondrogenesis study of hBMSC.

#### 2.5.1. Immuno-Histochemical (IHC) Analysis of Specific Protein Expressions of the Differentiations of Induced hBMSC to NP

IHC analysis of specific protein expressions the differentiations of hBMSC to NP after induced by TGF-β3 was carried out at 7 d and 14 d. All samples were washed using PBS and fixed by 4% paraformaldehyde. After the fixation, they were moved from the cultural wells and then embedded in paraffin and sectioned into sections with a thickness of 5 μm. The sections were then stained with Alcian blue for examining the depositions of glycosaminoglycan (GAG) and collagen type II, respectively. Cell-free hydrogel was stained as a negative control.

#### 2.5.2. Real-Time PCR for Specific Gene Expressions of the Differentiations of Induced hBMSC to NP

To determine the specific gene expressions of the differentiations of induced hBMSC to NP in the model hydrogels, the ECM-related gene expression, including Aggrecan (AGN), Collagen type I (Col I) and Collagen type II (Col II) were assessed by real-time PCR (StepOnePlus™ Real-Time PCR System, Applied Biosystems, Foster, CA, USA) at 7 d and 14 d, according to other studies [11,12,19]. GAPDH was used as the internal control. The relative gene expression was calculated as $2^{-\Delta \Delta C_{t}}$.

### 2.6. Statistics

All calculations are made using SigmaStat statistical software (Jandel Science, San Rafael, CA, USA) [10]. Statistical significance in the Student t-test corresponded to a confidence level of 95%. Data presented are mean ± SD from at least triplicate measurements. Differences were considered statistically significant at *p* < 0.05.

## 3. Results and Discussion

### 3.1. Fabricating Fluidity HA Hydrogels by Adjusting Parameters of HA, and Reactions Conditions of BDDE Crosslinking Reactions

HA hydrogels have high water contents because HA contents a large amounts of carboxyl groups and have been used for tissue repairs and in drug delivery systems [11]. However, without chemical modifications such as crosslinking of HA hydrogels, the gels would be easily disassembled in aqueous environment and then lost their mechanical properties which would usually deviate from those of human tissues including NP. Hydrogels for tissue repairs, including IVD repair, crosslinking HA to produce the HA network hydrogel has been widely prepared by oxidized HA or methacrylated HA, in order to improve and sustain the mechanical properties of HA [12,13,14]. However, it involves complex and delicate chemical reactions. Alternatively, HA network hydrogel was widely fabricated by crosslinking hydroxyl groups of *N*-acetyl-d-glucosamine (NAG) in HA using the epoxide groups of BDDE at a high pH condition (pH >11). In this study, HA network hydrogels were produced as the aforementioned method with modifying reaction parameters (Scheme 1). For the reactions in which HA was crosslinked by BDDE, several parameters such as the MW and concentrations of HA, the concentrations of BDDE, and the reaction time, would influence various properties of HA hydrogels [2,30]. For example, the uses of various concentrations of BDDE (0.01~20%) under alkaline conditions to crosslink various concentrations of HA with high molecular weight (2.65~10%, MW >10^3^ kDa) to produce HA crosslinked hydrogels have been extensively investigated [30]. Here, a low concentration (~2.5%) and a low MW (~200 kDa) of HA was crosslinked using around 2.0% BDDE for 5–7 h to produce HA crosslinked hydrogels (Scheme 1), which had low G′ and G″ (e.g., 0.24 ± 0.092 kPa and 0.09 ± 0.005 kPa, *n* = 3, respectively) with phase angle, δ, 21.4°, indicating that viscoelastic HA crosslinked hydrogels were high fluidity. Hence, HA crosslinked hydrogels could be mixed well with varying amounts of SF for producing HS-IPN hydrogels.

#### Using HRP/H_2_O_2_ Reactions to Crosslink SF and Producing HS-IPN Hydrogels

Although the de-sericin process for SF polymers affects its molecular weight [31], the de-sericin procedures herein were well controlled which ensured the molecular weight of SF was approximately 185 kDa, as determined by SDS-PAGE (data not shown). Since high fluidity of HA crosslinked hydrogels could be well mixed with SF, using HRP/H_2_O_2_ enzymatic reactions to crosslink Tyr in SF within the hydrogels could homogenously take place to produce SF-IPN hydrogels and, consequently, produce HS-IPN hydrogels (Scheme 1). To further stabilize the HS-IPN hydrogels, 0.15 mM of PEI added into the hydrogels was further crosslinked by EDC/NHS for about 30 min. to produce amide bonds of carboxyl groups of HA with amine groups in PEI and in SF in the hydrogels, respectively. (Scheme 1).

According to the results of rheological study (in Section 3.3), the viscoelastic properties of HS7-IPN hydrogels were similar to matrix for human NP. The HS7-IPN hydrogels were chosen as a model hydrogel for inducing hBMSC to differentiate to NP cells or NP tissue engineering in this study.

### 3.2. Characterizations of HS-IPN Hydrogels

#### 3.2.1. ATR-FTIR Spectra of HA, SF, and the Model Hydrogels Consisted of Varying Strains of SF

**Figure 1:**
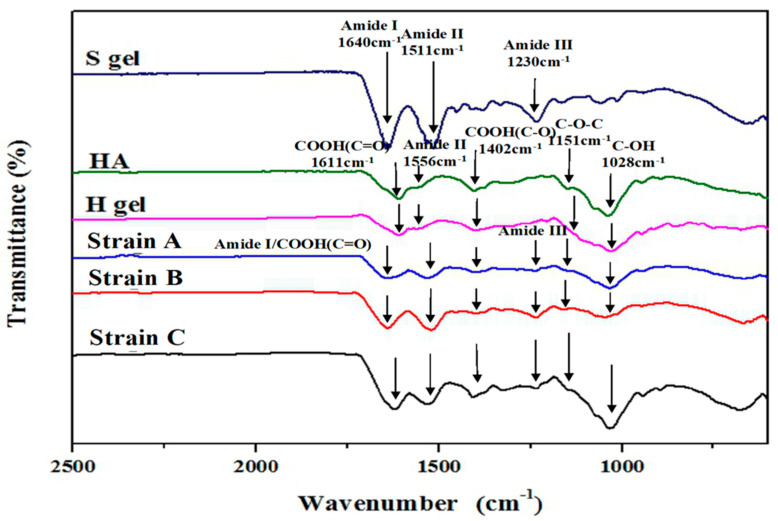
ATR-FTIR transmission spectra of varying functional groups of SF, HA polymers, and H, S and HS7-IPN hydrogels. The characteristic peaks of H hydrogels such as carboxyl groups (COOH), and S hydrogels such as amide I, II and III, respectively, were found in HS-IPN hydrogels without varying by the SF of strains of A, B and C.

The model hydrogels were characterized by spectra using an ATR-FTIR spectrophotometer (Figure 1). In the Figure 1, the transmission spectra for the peaks of carboxyl groups of H gels such as 1600 and 1402 cm^−1^ were about the same as those of HA polymers. Moreover, the peak of amide II of H gels was 1556 cm^−1^, which was similar to that of HA polymers. The transmission spectra for the peaks of amide I, II and III groups of S gels were 1640, 1511 and 1230 cm^−1^ that were about the same as SF polymers. The transmission spectra from HS-IPN gels produced from different strains of SF (e.g., strain A, B and C) contained several characteristic peaks as those of H gels and S gels with minor variations. For instance, the peaks of amide II of HS-IPN gels shifted from 1556 cm^−1^ of H gels to 1528, 1522 and 1525 cm^−1^ for strain A, B and C, respectively (Figure 1). The presences of the peak of amide I of HS-IPN hydrogels only minor shifted from 1640 cm^−1^ to 1635 cm^−1^ of S gels, and there was no difference among the model hydrogels consisted of varying strains of SF. Although the quantities of functional groups for varying weight ratios of HA/SF of the HS-IPN hydrogels were different such as HS1-IPN and the model hydrogels, the ATR-FTIR spectra for those hydrogels were similar to those of the model hydrogels and not able to characterize their differences (Figure 1). Hence, other characterizations including rheological properties of the hydrogels needed to be carried out to determine the differences among the model hydrogels consisted of varying strains of SF.

#### 3.2.2. The Pore Structures of Scaffolds and Swelling Ratios for H, S and the Model Hydrogels

**Figure 2:**
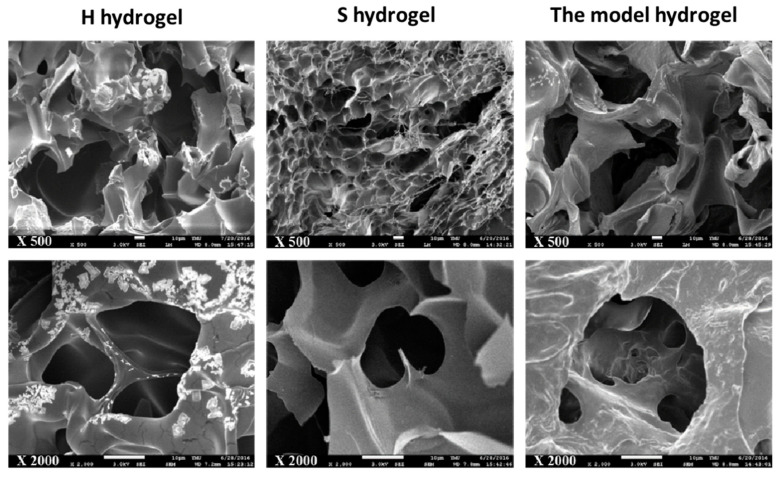
SEM micrographs of H, S, and HS-IPN hydrogels. The pore size of the model hydrogels was 38.96 ± 5.05 μm, (*n* = 3), in between those of H and S hydrogels.

To examine the pore structures of scaffolds for H, S and the model hydrogels, SEM micrographs of the scaffolds were carried out and presented (Figure 2). The mean pore sizes in H and S scaffolds were approximately 49.36 ± 15.04 μm, and 13.40 ± 1.25 μm, respectively, (*n* = 3). The mean pore size of the scaffolds of S gels was significantly smaller (*p* < 0.01, *n* = 3) than that for the scaffold of H gels (Figure 2). Interestingly, the mean pore size of the scaffolds of the model hydrogels was around 38.96 ± 5.05 μm (*n* = 3) which was between those H and S scaffolds. (Figure 2). In addition, the pore size of the scaffolds of the model hydrogels was suitable for the proliferations of cells, including hBMSC [7].

**Figure 3:**
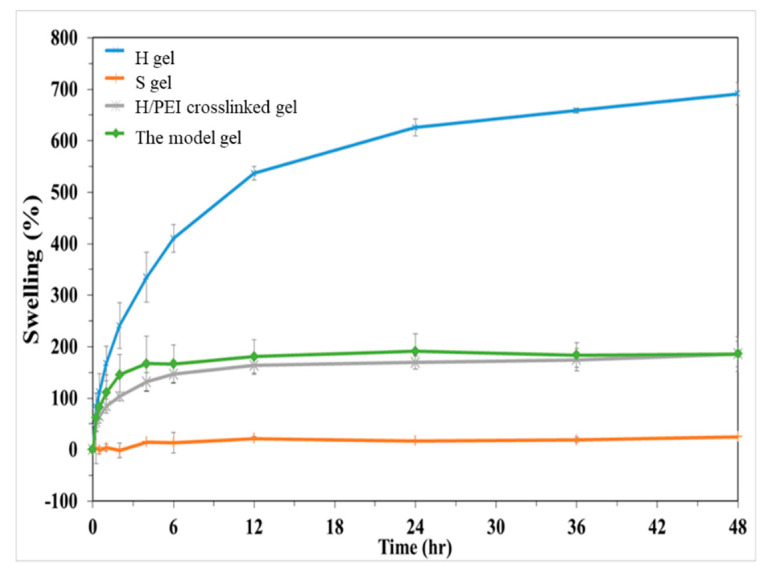
The swelling ratios for H, S, H/PEI crosslinked and the model hydrogels in PBS were shown. The swelling ratios for the H gels were significantly higher than others (*p* < 0.01, *n* = 3), while those for S gels were significantly lower than others (*p* < 0.01, *n* = 3). The swelling ratios of the model hydrogels in PBS were similar to those for crosslinking H/PEI crosslinked hydrogels between those of H and S hydrogels (Data are mean ± SD, *n* = 3).

The swelling ratios of H, S, H/PEI crosslinking and the model hydrogels in PBS were examined and shown (Figure 3). Since H gels were produced by highly hydrophilic polymers, the swelling ratios of the hydrogels fast increased within 12 h till 550% and then slowly increased up to ~700% at 48 h which was significantly higher than other hydrogels (*p* < 0.01, *n* = 3). Interestingly, after the first 6 h of fast swelling stage, the swelling ratios for the model and H/PEI crosslinked hydrogels were in plateau regions (e.g., 185.9 ± 24.4%, *n* = 3) until the end of study, 48 h. However, after swelling in the first 4 h, the swelling ratios for S hydrogels in PBS were about 24.9 ± 0.9% till 48 h, which were significantly lower than others (*p* < 0.01, *n* = 3) (Figure 3). The swelling ratios of the model hydrogels were very close to those of H/PEI crosslinked hydrogels, and the values were between those for H and S gels [32]. Notably, H/PEI crosslinked hydrogels highly reduced the swelling ratios in PBS compared with those for H gels, indicating that EDC/NHS reactions among HA and PEI in hydrogels would effectively crosslink carboxylic and amine groups to produce the amide bonds within the gels which resulted in significant decreases of the interactions among carboxylic groups of H gels and ambient H_2_O (Figure 3). Since EDC/NHS reactions were also carried out for preparing the model hydrogels as those for H/PEI crosslinked hydrogels, the swelling ratios for the model ones were similar to those of those for the later gels although the model hydrogels consisted of SF-IPN. In addition, the swelling ratios for other HS-IPN hydrogels (e.g., HS3-IPN hydrogels) would be similar to those for the model ones.

Notably, the results or data of HS-IPN gels at slow swelling ratio stages were similar to human NP, revealing that they may be suitable for use in TE of NP [7,28]. Moreover, the stability of the model hydrogels in PBS solution was examined by observation and measuring weight loss of the hydrogels after they were immersed into PBS solution for three weeks. The morphology of the model hydrogels was intact without disintegration by observation while approximately 4% of weight loss was found compared to that of the original hydrogels to be immersed in PBS. In addition, the samples became more fluidity towards the end of the four weeks. Notably, the results of HS-IPN gels at slow swelling ratio stages were similar to human NP, revealing that they may be suitable for use in TE of NP [28].

### 3.3. Varying Strains of SF Influenced the Rheological Properties of HS-IPN Hydrogels

Viscoelastic flow parameters are important mechanical properties to characterize hydrogels for applications of bio-fluids such as lubricant in joints or NP. To determine, G′, G″ and δ values of HS-IPN hydrogels, they are determined using a parallel-plate rheometer operated in an oscillatory mode at a fixed strain (0.01 rad) with various angular frequencies (1–100 rad/s) as used by other studies [11,12,28]. G′ of the model hydrogels consisted of SF of strain A was 4.09 ± 0.32 kPa (*n* = 3, *p* < 0.01) which was significantly higher than those consisted of SF of strains B and C at a fixed angular frequency (Table 1). Since strain A of SF consists of more amount of Tyr than those in strain B and C, it could be assumed that more di-tyrosine bones were formed to crosslink peptides of strain A of SF to produce SF-IPN hydrogels than those in B and C ones. Hence, the rheological properties of the HS-IPN hydrogels were influenced by the strain of SF. The results rheological properties influenced by strains of SF, presented in Table 1, were qualitatively consistent with those UV-excited fluorescent intensities presented in Table 2. According to amino acid analysis for three strains of SF in this study, the total number of amino acids were about 5500, including about 5.1% of Tyr in strain A, while about 4.6% of tyrosine in strains B and C. The Tyr contents for strains of B and C were similar to other reports [27]. Notably, the formations of varying amounts of dityrosine bonds in SF-IPN hydrogels, crosslinked by HRP/H_2_O_2_ would emit varying intensities of blue fluorescence when the hydrogels were irradiated by UV [29]. The intensity of emitted blue fluorescence of SF-IPN hydrogels for strain A was significantly higher (*n* = 4, *p* < 0.01) than those of strains of B and C (Table 2), which was consistent with that Tyr contents in strain A (e.g., 277 ± 11, *n* = 4) is significantly higher than those of strain B and C (e.g., 255 ± 2, *n* = 4 for B), respectively. Therefore, the influences of strains of SF on the rheological properties of HS-IPN hydrogels possibly resulted from the tyrosine contents in each strain of SF.

#### Influence of the Weight Ratios of SF to HA in HS-IPN Hydrogels on Rheological Properties of Hydrogels

**Figure 4:**
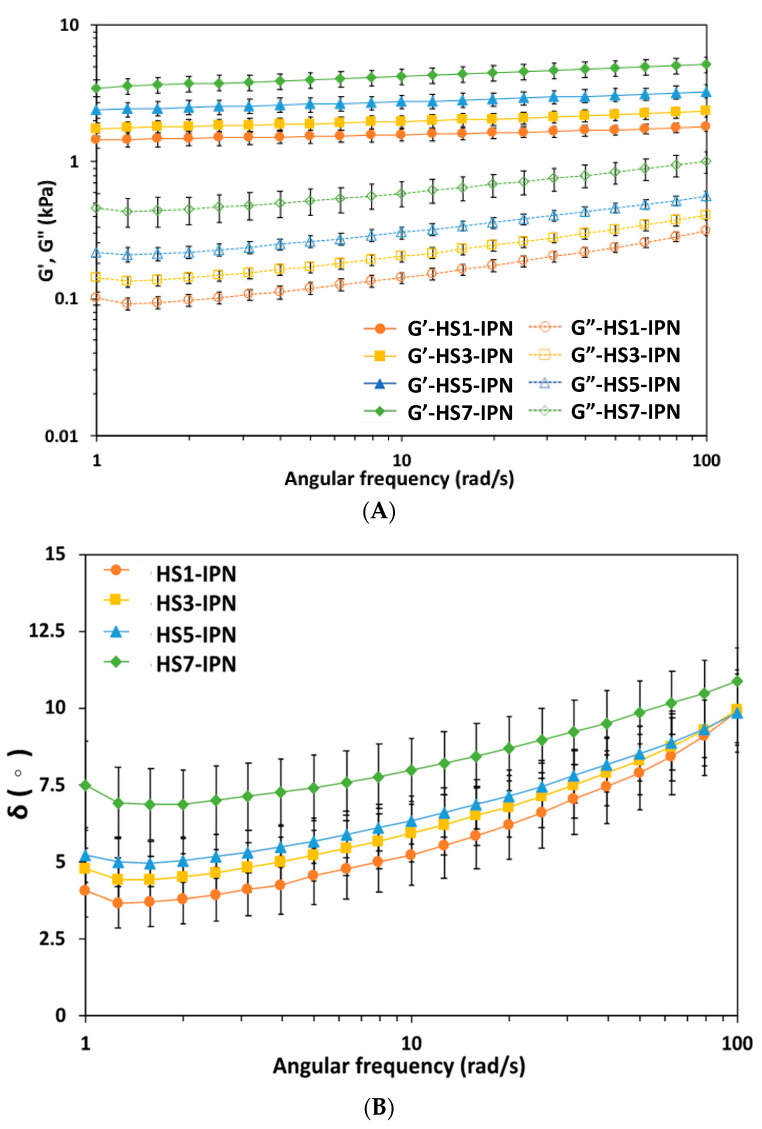
(**A**) The rheological parameters (e.g., G′ and G″) for all tested HS-IPN hydrogels increased with increasing the weight ratios of SF/HA in producing the hydrogels versus the varying angular frequencies, (**B**) increasing weight ratios of SF/HA in the HS-IPN hydrogels increased the δ values from about 4.2 (e.g., HS1-IPN) hydrogels to 8.0° (e.g., HS7-IPN) hydrogels vs. with varying angular frequencies, respectively (Data are mean ± SD, *n* = 3).

Other than the SF strains, the weight ratios of SF to HA in producing the HS-IPN hydrogels might also influence the viscoelastic properties of the hydrogels. For examining this factor, at a fixed HA content (4%), varying SF concentrations (in wt.%) in producing HS1-IPN to HS7-IPN hydrogels (or the model gels) were carried out at the aforementioned oscillatory conditions (Figure 4A). G′ values increased with increasing the concentrations of SF in the HS-IPN hydrogels. Therefore, the model hydrogels had the highest G′ and G″ values (*p* < 0.001, *n* = 3) among the produced hydrogels herein tested at varying angular frequencies. For example, the G′ values of the model hydrogels (e.g., 4.09 kPa at 10 rad/s) were about 2.6 times higher than those of HS1-IPN gels.

The phase angles, δ values, for all HS-IPN hydrogels were shown (Figure 4B) which increased from around 4.2° to 8.0° at 10 rad/s, respectively, (*n* = 3). The results of δ values indicated that the model hydrogels were less viscoelastic solid than others (e.g., HS1-IPN), respectively. In comparison, the rheological properties for HA crosslinked hydrogels, produced by the same protocols as those for HS-IPN hydrogels, were carried out and had low G′ (e.g., 0.24 ± 0.092 kPa, *n* = 3) with phase angle of 21.4°. The results indicated that viscoelastic properties for HA crosslinked hydrogels were high fluidity with a very low elastic modulus. Hence, the results of rheological properties for HA crosslinked hydrogels were not suitable for TE of NP. Notably, the G′ and δ values at 10 rad/s for the HS7-IPN hydrogels produced herein were similar to those reported for native NP (5.0~10.3 kPa and 2.5°~35°, respectively) [7,18,28], and therefore, the hydrogels were selected as the model hydrogels for further this investigation.

The G′ for IPN hydrogels produced herein were similar to those of laminin-111-PEG hydrogels reported for the matrix of NP [24]. Interestingly, the rheological modulus for G′ or |G*|of HS1-IPN hydrogels were fitted the requirements of hydrogels for cardiac repairs. According to the tyrosine contents of varying strains of SF were different (Table 2), and the strains of SF influenced the viscoelastic modules of HS-IPN hydrogels (Table 1). The contents of tyrosine of SF were one of an important factor on determining those modules of HS-IPN hydrogels although the phase angles of the hydrogels might not be the case as those modules (Table 1). Moreover, the model hydrogels contained more concentrations of SF and amounts of di-tyrosine bonds in qualitative in the hydrogels than other HS-IPN hydrogels that resulted in increasing their viscoelastic modulus (Figure 4A and Table 1). However, the amounts of di-tyrosine bonds in each HS-IPN hydrogel were not able to be determined quantitatively. Although, the bonds could be semi-quantitatively evaluated using the intensity of UV-excited blue fluorescence [29].

Although using NHS/EDC to crosslink HA/SF to facilely prepare HA/SF hydrogels was recently reported [23], it was only one step of our processes to prepare hydrogels. However, they did not perform the rheological study for the aforementioned hydrogels [23]. Recently, using sonication and UV photo-polymerization to prepare SF/methacrylated HA or to produce SF-based IPN hydrogels has been reported by Xiao et al. [33], respectively. However, the rheological properties of the hydrogels were not determined. Interestingly, using HRP/H_2_O_2_ to crosslink tyramine-substitute HA and 2% of SF to produce hydrogels with varying HA contents has been reported by Raia et al. [24]. HA and SF-IPN hydrogels produced in their study are configured by di-tyramine bonds in HA crosslinked network, di-tyrosine bonds in SF crosslinked network and tyramine-tyrosine bonds in HA/SF-IPN, which bonding structures of their hydrogels were distinct from those in HS-IPN hydrogels produced in this study. Hence, the rheological properties of our hydrogels were different from theirs [24]. Moreover, the tyramine-substitute HA needed to be complexly and chemically synthesized for the research which was not a commercially available biomaterial, while the biomaterials were generally commercially available.

### 3.4. Confined Compressive Modules of the Model Hydrogels

The confined compressive stress of the model hydrogels was conducted on the hydrogels under 5% strain with value of 0.109 ± 0.011 MPa (*n* = 3) which was slightly lower than the for human NP (e.g., around 0.5–1.5 MPa) [7]. Notably, the compressive modulus for the model hydrogels was 2.29 ± 0.05 MPa (*n* = 3) was similar to that of human NP [7]. Although using NHS/EDC to crosslink HA/SF to facilely prepare HA/SF hydrogels was recently reported by Yang et al. [23] that was generally simple to produce the hydrogels than those produced by the procedures for this study, the confined compressive stress of their products was about 12 kPa at 30% of strain, which was about 25 times less than the stress of our model hydrogels (e.g., 314 ± 2.8 kPa, at 15% strain, *n* = 3). Hence, the compressive stress for their hydrogels would not fit the need of human NP.

Recently, Xiao et. al. [34] reported that using sonication and further UV photo-polymerization to prepare SF-IPN and methacrylated HA to produce network SF/HA hydrogels, which had a stiff but brittle SF structure while SF-IPN hydrogels produced herein (Scheme 1) were non-brittle. However, the confined compression modules of their hydrogels were not examined [23,31] as did in this study.

### 3.5. Cytotoxicity Examinations for the Model Hydrogels

**Figure 5:**
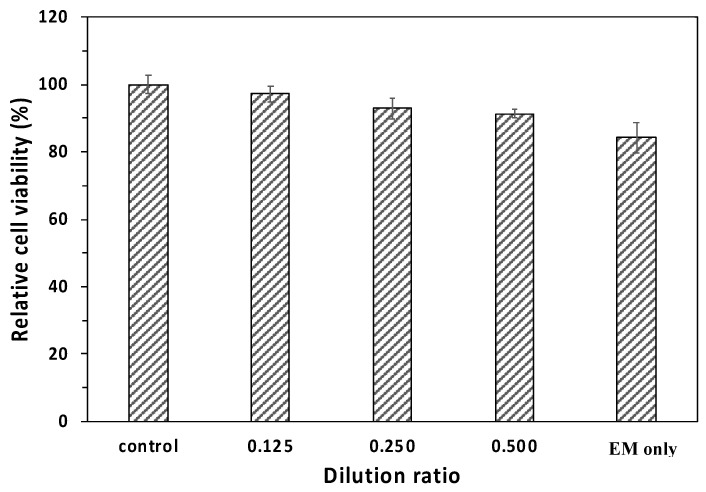
L929 Cell viability incubated by the extraction mediums (EM), taken from supernatants which was incubated with the model hydrogels for 24 h, at different dilute ratios according to the guidelines of ISO 10993-5. The relative L929 viability (%) of the group with a dilution ratio of 0.5 exceeded that of the group of EM only. The results of MTT assay for L929 Cell viability showed that the model hydrogels were biocompatible biomaterials (data are mean ±SD, *n* = 3).

In-vitro cytotoxicity for the model hydrogels were performed according to the requirements of ISO10993-5. Briefly, after L929 cells were cultured with vary concentrations of extraction mediums, for 24 h, and the MTT assay to the cells were carried out to examine the cell viabilities [26]. According to the MTT assay shown in Figure 5, the L929 viability of the group with a dilution ratio of 0.5 exceeded that of the group of extraction medium only which met the requirements of ISO10993-5, revealing that the model hydrogel was biocompatible and suitable for use in TE. The results of the biocompatibility of the hydrogels were similar to other SF-based biomaterials [8,31].

### 3.6. Differentiations of hBMSC to NP Cells in the Model Hydrogels

**Figure 6:**
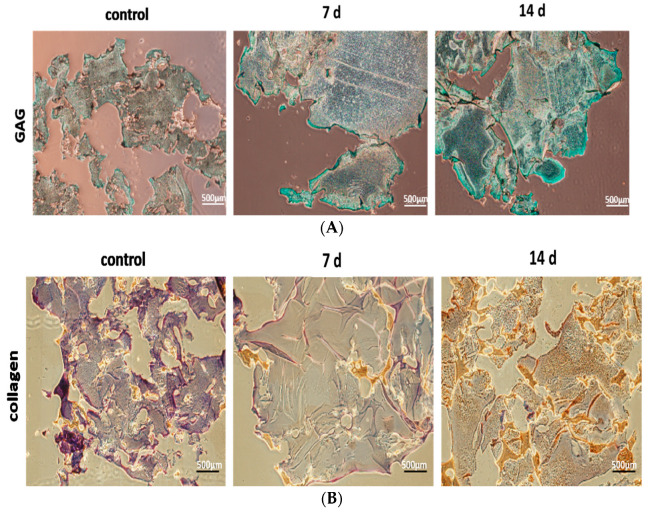
Immuno-histochemical (IHC) analysis of hBMSCs in the model hydrogels after hBMSC were induced by TGF-β3 to differentiate them to NP cells for 7 d and 14 d, respectively. Deposition of (**A**) GAG (in blue) and, (**B**) Collagen type II (in brown) in the model hydrogels were shown which were significantly higher than those of cultivated in cultural wells (e.g., control group).

To evaluate the differentiation of hBMSCs to NP cells, they were induced by TGF-β3 in the model hydrogels. The morphology of hBMSC, and the accumulations of NP-related ECM deposits in the hydrogels were examined using vimentin stain and immuno-histochemical (IHC) analysis, such as glycosaminoglycan (GAG) and collagen type II stain, respectively. The cell cultivated in cultural wells was also stained as a control group. Figure 6A exhibited that the deposition of glycosaminoglycan (GAG, in blue), one of the main ECMs in the NP, in hBMSC-laden model hydrogels was much more than that in the control [34]. Collagen type II, an important component of ECM in NP tissue, forms a fibrillar network that traps proteoglycan and resists swelling [11,12,20]. The deposition of GAG (in blue) and accumulation of collagen type II (in brown) in the model hydrogels containing differentiated hBMSC increased with increasing the cultural period (Figure 6A,B, respectively). The depositions of the GAG and collagen type II were broad distribution in the model hydrogels, revealing that the hydrogels herein were suitable to the differentiations of hBMSCs to NP cells. Enhancing expressions in GAG and collagen type II of the differentiations of hBMSCs to NP cells in the model hydrogels compared to those expressions in the control group revealed that they promoted the differentiations of hBMSC (Figure 6A,B, respectively). According to those results, the mechanical properties of the model hydrogels compared with hardness matrix (i.e., cultural wells) were suitable to the differentiations of hBMSCs to NP cells.

**Figure 7:**
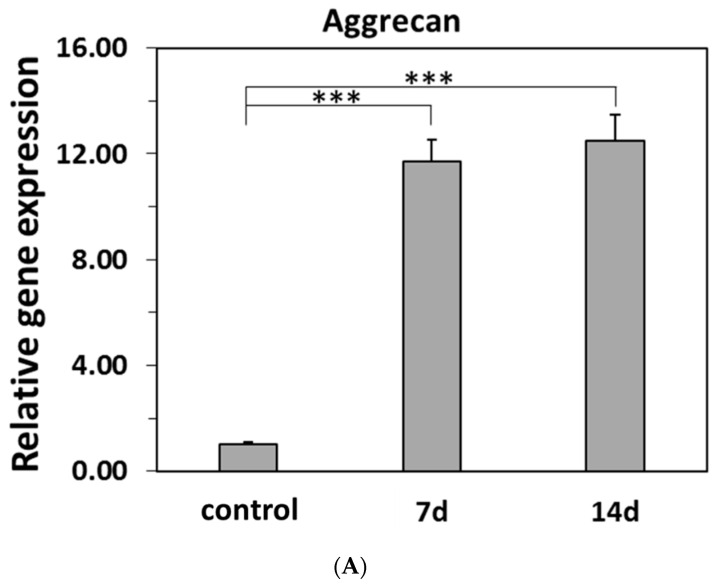

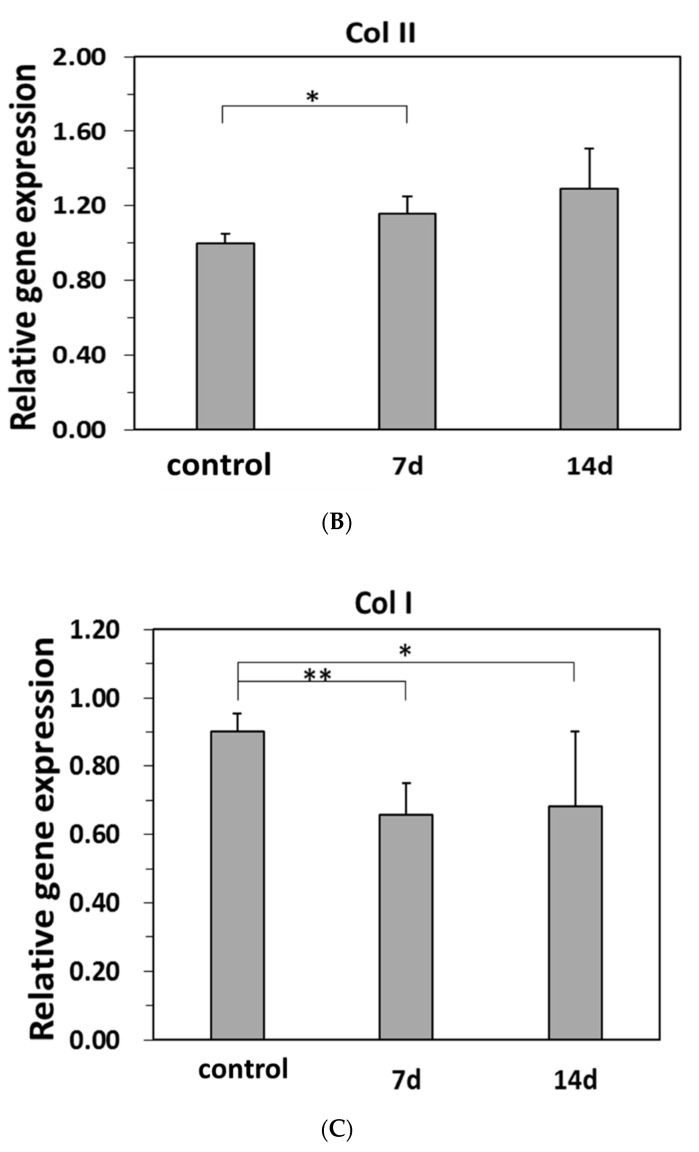
Real-time PCR analysis for the relative quantities of NP-specific gene expressions of inducing differentiation of hBMSC to NP cells on the model hydrogels for 7 d and 14 d. Gene expressions of AGN (**A**), COL II (**B**) and COL I (**C**) for the control group (e.g., ctrl) were the data for 7 d incubated at cultural wells. (* *p* < 0.05, ** *p* < 0.01, ***p < 0.001; data presented are mean ±SD, *n* = 3).

Aggrecan (AGN), collagen type II (Col II) and collagen type I (Col II) were selected as test genes for examining the differentiations of hBMSCs in the model hydrogels [11,12,21]. The gene expressions of chondrogenesis of hBMSCs, cultured in the model hydrogels, revealed significant up-regulation of both AGN and Col II but significant down-regulation of Col I, compared to those of hBMSCs cultivated in cultural wells (e.g., the control group) for 7 d (Figure 7A–C). The results gene expressions of differentiations of hBMSCs to NP cells presented herein were similar to those of other studies [11,12,13] although the compositions of their hydrogels were different from this study. AGN and Col II gene expressions increased with the 7 d of differentiations of hBMSCs to NP cells in the model hydrogels that were consistent with the increasing depositions of GAG and collagen type II of IHC stains (Figure 6A,B, respectively). However, the gene expressions of the differentiations of hBMSCs to NP cells in the model hydrogels (Figure 7A–C, respectively) were not further increased in the period of 7 d to 14 d. The possible mechanisms such like reducing the activity of CD44 of HA for the differences of results the differentiations of hBMSCs to NP cells in Figure 6 and Figure 7 at 14 d need to be further studied. Although adjust mechanical properties of HA-based or methacrylated HA-based containing SF hydrogels using varying techniques for other TE studies have been reported by others [11,23,24]. This study reported an examination of the differentiations of hBMSCs to NP cells in HS-IPN hydrogels. Nevertheless, the model hydrogels promoted the differentiations of hBMSCs to NP cells for tissue engineering of NP.

## 4. Conclusions

The roles of SF on characteristics of HS-IPN hydrogels were examined. The schematic procedures for preparing HS-IPN hydrogels were presented in Scheme 1. The pore size of the model hydrogels was 38.96 ± 5.05 μm between those of H and S hydrogels (Figure 2). The swelling ratios for HS-IPN hydrogels (e.g., 185.9 ± 24.4%, *n* = 3) were similar to those for H/PEI hydrogels (Figure 3) which were much less than those for HA hydrogels. The hydrogels composed of SF of strain A showed higher G′ and G″ values than those composed of SF of strain B and C (Table 1 and Table 2, respectively). Moreover, the model hydrogels consisted of the highest weight ratios of SF to HA showed significantly higher G′, G″ and |G*|values than other hydrogels consisted of those of low weight ratios. (Figure 4A). The model hydrogel was biocompatible for TE applications (Figure 5). Moreover, the model hydrogels significantly promoted the differentiations of hBMSC to NP cells with increasing the expressions of GAG and collagen type II for 7 d and 14 d (Figure 6). Moreover, the induced hBMSC in the hydrogels increased the gene expressions of AGN and COL II while decreased those of COL I for 7 d of cultivations (Figure 7). Hence, the model hydrogels developed herein were suitable to tissue engineering for NP regeneration.

## Figures and Tables

**Table 1:** The viscoelastic parameters of the model hydrogels fabricated from different strains of SF measured at 0.01 rad and 10 rad/s (*n* = 3). The parameters of strain A such as G′ and |G*|, complex shear modulus, were significantly higher than strain B and C.

Materials	Viscoelastic Properties (0.01 Rad, 10 rad/s)			
	G′ (kPa)	G″ (kPa)	|G*| (kPa)	δ (°)
Strain A	4.09 ± 0.32 **	0.59 ± 0.16	4.13 ± 0.34 **	8.14 ± 1.66
Strain B	3.24 ± 0.16	0.50 ± 0.05	3.27 ± 0.16	8.69 ± 0.5
Strain C	3.40 ± 0.19	0.43 ± 0.07	3.43 ± 0.18	7.28 ± 1.19

** *p* < 0.01 or better.

**Table 2:** The intensity of blue fluorescence of SF hydrogels excited by UV irradiation, in terms of OD values, for different strains of SF at 1.62 mM H_2_O_2_ [29]. (*n* = 4).

The Intensity of Blue Fluorescence of SF Hydrogels Consisted of Vary Strains of SF with Their Tyrosine Contents		
Sample	OD Value	Tyrosine Content
Strain A	0.80 ± 0.01 **	277 ± 11 **
Strain B	0.67 ± 0.01	255 ± 2
Strain C	0.67 ± 0.02	255 ± 1

** *p* < 0.01.

## Nomenclature

Nomenclature	Full name
HA	Hyaluronic acid
SF	Silk fibroin
H gel	HA hydrogel
S gel	SF hydrogel
HS1-IPN hydrogel	HA/SF hydrogel with weight ratios of HA to SF was 5:1
HS3-IPN hydrogel	HA/SF hydrogel with weight ratios of HA to SF was 5:3
HS5-IPN hydrogel	HA/SF hydrogel with weight ratios of HA to SF was 5:5
HS7-IPN hydrogel (the model hydrogel)	HA/SF hydrogel with weight ratios of HA to SF was 5:7
